# Prevalence, content and significance of advance care planning in nursing home patients

**DOI:** 10.1080/02813432.2022.2036429

**Published:** 2022-02-16

**Authors:** Lisa Kastbom, Magnus Falk, Marit Karlsson, Anders Tengblad, Anna Milberg

**Affiliations:** a Department of Health, Medicine and Caring Sciences, Linköping University, Linköping, Sweden; b Primary Health Care Centre in Kisa, and Department of Health, Medicine and Caring Sciences, Linköping University, Linköping, Sweden; c Primary Health Care Centre in Kärna, and Department of Health, Medicine and Caring Sciences, Linköping University, Linköping, Sweden; d Department of Biomedical and Clinical Sciences, Linköping University, Linköping, Sweden; e Department of Advanced Home Care in Linköping, and Department of Health, Medicine and Caring Sciences, Linköping University, Linköping, Sweden; f Primary Health Care Centre in Wetterhälsan, Jönköping, Sweden; g Department of Advanced Home Care in Norrköping, and Department of Health, Medicine and Caring Sciences, Linköping University, Linköping, Sweden

**Keywords:** Advance care planning, end-of-life care, nursing homes, palliative care, primary healthcare

## Abstract

**Objective:**

Studies on advance care planning in nursing homes are rare, and despite their demonstrated favourable effects on end-of-life care, advance care plans are often lacking. Therefore, we wished to explore: (i) the prevalence of advance care plans in a Swedish nursing home setting using two different definitions, (ii) the content of advance care plans, (iii) adherence to the content of care plans and (iv) possible associations between the presence of advance care planning and background characteristics, physician attendance and end-of-life care.

**Design:**

Retrospective chart review.

**Setting:**

Twenty-two nursing homes in Sweden.

**Subjects:**

A total of 367 deceased patients (included between 1 June 2018 and 23 May 2020) who had lived in nursing homes.

**Main outcome measures:**

Electronic health record data on the prevalence of advance care plans with two different definitions and variables regarding background characteristics, physician attendance and end-of-life care, were collected.

**Results:**

Of the study population, 97% had a limited care plan (ACP I) documented. When using the comprehensive definition (ACP II), also including patient’s preferences and involvement of family members in advance care planning, the prevalence was 77%. Patients with dementia more often had care plans, and a higher physician attendance was associated with presence of advance care plans. Prescription of palliative drugs and information to family members of the patient’s deterioration and impending death were more common in patients with care plans compared to those where such plans were missing. There was adherence to the care plan content.

**Conclusion:**

In contrast to previous research, this study showed a high prevalence of advance care plans in nursing home patients. Patients with care plans more frequently received prescriptions of palliative drugs and their family members were informed to a greater extent about the patient’s deterioration and impending death compared to those without care plans. These aspects are often seen as vital components of good palliative care.

**Key points**
- Studies on advance care planning in nursing homes are rare, and despite their demonstrated positive effects on end-of-life care, advance care plans are often lacking.
- The present study revealed a high prevalence of advance care plans (77-97% depending on definition) in nursing home patients.
- Patients with dementia more often had advance care plans, and a higher physician attendance was associated with presence of care plans.
- Advance care plans were positively associated with components of good palliative care, such as prescriptions of palliative drugs and information to family.

## Introduction

Since nursing home patients are often old and frail [1], most deaths in nursing homes are expected deaths. Therefore, making proactive plans for future care, that is, advance care planning, should be seen as central, in order to create conditions for good end-of-life (EOL) care and a good death [2].

Previous studies have shown positive effects of advance care planning in EOL care, such as reduced hospital admissions, decreased number of days spent in hospital, place of death in accordance with patient preferences and positive economic aspects [3–7]. Detering et al. showed that advance care planning in elderly hospitalised patients was associated with improved quality of life and reduced aggressive/intensive care at EOL [3]. In a systematic review of the effects of advance care planning in nursing home patients, care planning was shown to decrease hospitalisation and increase the proportion of patients dying in their nursing home, rather than in hospital. Medical treatments in accordance with patient preferences were also more frequent with advance care planning [7].

Although many elderly people wish to participate in advance care planning [1,3,8–12], EOL discussions, which are crucial elements of care planning, are reported to be rare [1,8–10,13–17]. Sharp et al. showed that only 2–29% had discussed EOL care plans with healthcare staff [10]. In an Australian study, only 0.2% of nursing home patients had a care plan [13]. The practice of care planning is well established in Sweden. Flo et al. concluded in a review that studies on advance care planning in nursing homes are rare and that there are variations in the definitions and content of advance care planning [18]. In the various definitions of advance care planning, a decision-making process is often part of the content [19–21]. Some also highlight the aspect of preparing the patient and family members for EOL [19–21]. Aspects frequently present in advance care planning are proactive planning for future care, patient preferences concerning treatment and care, and involvement of family members. These aspects are central in the EAPC (European Association of Palliative Care)-supported consensus-based definition of advance care planning [19]. In Sweden, a decision to shift focus from any level of life-prolonging or life-sustaining care to strictly palliative care is a widely used strategy, referred to as ‘breakpoint decision’ [22] and equals a palliative care plan.

The prevalence of advance care planning in nursing home patients in Sweden is unknown, as well as its possible associations with the quality and content of EOL care for these patients. Therefore, the aim of this study, conducted in a Swedish nursing home setting, was to explore (i) the prevalence of advance care plans (using two different definitions), (ii) the content of advance care plans, (iii) adherence to the content of care plans and (iv) possible associations between the presence of advance care planning and background characteristics (e.g. demographic and diagnosis), physician attendance at the nursing home and EOL care.

## Material and method

This study was performed as a retrospective chart review. The deceased patients were included from 22 nursing homes in two Swedish counties during two years (between 1 June 2018 and 23 May 2020). During the study, the Covid-19 pandemic began (pandemic outbreak in March 2020). Primary-care physicians in some counties were encouraged to establish advance care plans concerning serious respiratory-tract infections in nursing home patients during the pandemic.

### Clinical setting

In Sweden, an increasing number of older people live in their own homes, rather than nursing homes, which has contributed to a situation where people moving into nursing homes are usually the oldest and most frail, with multimorbidity. Approximately 20% of the patients die within six months from moving into the nursing home [23]. Nursing home care in Sweden is provided by two authorities in cooperation: regions and municipalities. Each nursing home has attending physicians employed at a regional primary healthcare centre, usually general practitioners (GPs) or general practitioner specialist trainees (GP-STs). Nurses and other staff are municipality employees. This means that the physicians and nurses working with nursing home patients have different employers, and also use different patient health record systems. These documentation systems are not compatible, and nurses and physicians cannot access each other’s systems. The nursing homes included in the present study were long-term care homes, including care homes for patients with dementia. Units for short-term care only (care homes for patients waiting to be transferred to long-term care homes or to ordinary homes after being discharged from hospital) were not included in this study. The attending physicians were employed at ten different primary healthcare centres.

### Study population

In County A, a data search was performed using a municipality healthcare administrative system database (Treserva, CGI) to identify deceased patients who had lived in any of the 14 nursing homes in the municipality. In County B, a data search was performed in the county's electronic health record (Cambio Cosmic), identifying deceased patients who had lived in any of the eight nursing homes connected to the two primary healthcare centres selected during the inclusion interval. Inclusion criteria were: deceased nursing home patients having lived in any of the selected nursing homes during their last days of life during the inclusion interval. Both rural and urban located nursing homes in the two counties were represented and the nursing homes had possibility to accommodate 7–180 patients.

### Health record review

In total, 367 electronic health records were analysed. LK (first author of this paper), being a GP, performed the retrospective chart review in 2020. In the health record review, data on the prevalence of advance care plans were collected, as well as variables regarding background characteristics, physician attendance (number of physician consultations at the nursing home during the final six months of the patient’s life) and EOL care, consisting of: information given to family members about the patient’s deterioration and impending death, emergency department (ED) visits and instances of inpatient care during the patient’s final six months of life, prescription of palliative drugs for symptom relief, prevalence of “benefit for care of closely related person” certificate (an employee's right to be off work in order to attend a closely related person who is seriously ill and receive economic compensation through Swedish social insurance) [24]. Adherence to the content of advance care plans was explored by studying the associations between a documented instruction to limit non-beneficial hospital care and frequency of ED visits or inpatient care. Limitations on hospital care in the care plans were concluded to be written instructions in the health record to limit hospital care, in favour of care at the nursing home, such as: patient should never receive hospital care, or hospital care only in case of fracture, acute chest pain, etc.

In a pilot sub-study, the data-collection protocol (Appendix) and coding were evaluated. One co-author (MK) recoded a subsample of the health records (*n* = 10). There was no disagreement about the coding of these 10 health records. During the further retrospective chart review, performed by LK, a few additional health records were discussed with MK when there were difficulties or uncertainty in the interpretation and understanding of the data extraction, to reach consensus.

### Definition of advance care planning

Based on the existing accepted definitions of advance care planning [19–21], the description of the Swedish term in the National Board of Health and Welfare (Swedish: Socialstyrelsens termbank) [22] and clinical experiences of the authors, one limited definition (ACP I) was concluded as ‘a proactive plan to handle a future deterioration of the patient, not only to handle a current, specific problem or situation’. Inspired by the consensus definition of advance care planning, supported by EAPC [19], a comprehensive definition was also developed (ACP II). ACP II included the definition of ACP I, and also: (i) the presence of the patient’s preferences concerning medical treatment and care (if capable of expressing them, meaning that patients with cognitive impairment did not have to fulfil this criterion of the ACP II definition), and (ii) involvement of family members in the advance care planning. In the present study, a ‘breakpoint decision’, that is a palliative care plan [22], described in the introduction, was considered to be an advance care plan. However, care plans concerning only serious respiratory-tract infections, were not considered to fulfil the ACP I or the ACP II criterion, as such plans were concluded as proactive plans to handle a future deterioration of the patient restricted to a single, specific problem.

### Statistics

Categorical data variables were presented as numbers (*n*) and proportions (percentages), and continuous variables as medians and means. Pearson’s chi-square test was used when comparing the distribution of proportions between groups, and Fischer’s Exact test when the expected number was less than five in any cell. For group comparisons of continuous variables, Mann–Whitney U-test was used, since the data were considered not to be normally distributed. *P* values <0.05 were considered statistically significant. IBM SPSS Statistics 27 (IBM Corporation, NY, USA) and Excel were used for statistical analyses.

## Results

Median length of stay at the nursing home was 26 months (range 0–140) and of the total study population (*n* = 367), about two-thirds were women. Prevalence of diagnoses in the patients are shown in Table 1. Eighty-six percent died in their nursing home, while 14% died in hospital.

**Table 1.** Prevalence of diagnoses in the total study population (*n* = 367).

Diagnosis	Prevalence (*n*, %)
Cardiovascular disease	274 (75%)
Diabetes	72 (20%)
Dementia	225 (61%)
Pulmonary disease	51 (14%)
Cancer disease	47 (13%)
Stroke	82 (22%)
Psychiatric disease	132 (36%)
Kidney failure	63 (17%)

### Prevalence and content of advance care plans

The great majority of the study population (*n* = 355; 97%) had at least one advance care plan, where the definition of ACP I was fulfilled. ACP II, that is, the comprehensive definition, was present in 77% (*n* = 282) of the individuals. Care plans restricted to serious respiratory-tract infections during the Covid-19 pandemic were present in 74% (35 of 47 deaths) and 17% (5 of 29 deaths) respectively in the two counties represented between middle of March 2020 to the end of the inclusion period in May 2020. Advance care plans that included palliative care plans (“breakpoint decisions”) were documented in 86% of the study population. In 15 of the 52 patients without such a palliative care plan, death occurred suddenly or without obvious deterioration before death. The advance care plans in the health records were written either in templates or in linear format. Median time from move into nursing home to first care plan was eight months, while most of the nursing home patients had their last plan documented during their last month of life (range 0-37 months before death). Table 2 illustrates the content of the advance care plans in the deceased patients’ health records.

**Table 2.** Overview of the content of the advance care plans in the deceased patient’s health records. Advance care planning according to the limited definition (ACP I) was identified in 355 of the total study population of 367.

Variable	Number (%)	Median (range)	Mean (SD)
Number of ACPs per patient		2 (0–7)	2.2 (1.1)
Time from move into NH to first ACP (months)		8 (0–120)	18.5 (23.7)
Time from first ACP at NH to death (months)		8 (0-119)	16.3 (21.0)
Time from last ACP to death (months)		0 (0–37)	1.3 (4.5)
**Patient’s preferences^a^ documented**			
Patient’s preferences present	117 (33%)		
Patient’s preferences missing, no cognitive impairment	34 (10%)		
Patient’s preferences missing, cognitive impairment	204 (57%)		
**Family members involved in ACP**			
Family members participating^b^ in ACP	239 (67%)		
Family members informed of ACP content	305 (86%)		
Do-not-resuscitate order (DNR)	297 (81%)		
Hospital care limitation^c^	265 (72%)		

^a^Patient’s preferences concerning direction in care and care limitations. ^b^Family members participating in ACP, physically or by phone. ^c^A written instruction in the ACP document to limit hospital care, such as: patient should never receive hospital care, or hospital care only in case of fracture, acute chest pain etc.

### Adherence to the content of advance care plans

Among individuals with hospital care limitations specified in their advance care plans, both ED visits and inpatient care were significantly less frequent, compared to those who lacked such limitations (Table 3). The most common reasons for ED visits in the 66 individuals of the study population despite hospital care limitations were infections (*n* = 23), hip fractures (*n* = 10), dyspnoea (*n* = 8), trauma from falls (*n* = 4), suspected stroke (*n* = 4), abdominal pain (*n* = 3) and cardiac failure (*n* = 2). We could not see an increase in admissions during the Covid-19 pandemic.

**Table 3.** Adherence to hospital care limitations^a^ in the care plan (two different definitions: ACP I and ACP II) were evaluated by using frequency of ED visits and inpatient care during the patient’s final six months of life.

Variable	ACP I and hospital care limitations (*n* = 265)	ACP I and no hospital care limitations (*n* = 90)	*p* value	ACP II and hospital care limitations (*n* = 218)	ACP II and no hospital care limitations (*n* = 64)	*p* value
≥ One ED visit(s) during the patient’s final six months of life (n, %)	66 (25%)	50 (55%)	*<0.001*	53 (24%)	35 (55%)	*<0.001*
≥ One period of inpatient care during the patient’s final six months of life (n, %)	52 (20%)	48 (53%)	*<0.001*	40 (18%)	35 (55%)	*<0.001*

Pearson’s chi-square test was used for group comparisons of categorical data. *p* values <0.05 were considered significant. ^a^Written instructions in the ACP document to limit hospital care, such as: patient should never receive hospital care, or hospital care only in case of fracture, acute chest pain etc.

### Associations between the presence of advance care planning and background characteristics, physician attendance and EOL care

There were associations between advance care planning and background characteristics, physician attendance and EOL care (Table 4). Dementia was significantly more common among patients with ACP II than in those without ACP II, whereas the opposite relationship was seen for cardiovascular disease and kidney failure. Physician consultations at the nursing home during the final six months of the patient’s life were significantly more common in individuals with ACP I than without a care plan. Both ACP I and ACP II were positively associated with information being given to family members of the patient’s deterioration and impending death, as well as prescription of palliative injections prior to death.

**Table 4.** Overview of associations between advance care planning and background characteristics, physician attendance at nursing home and EOL care.

	No ACP I^a^*n* = 12	ACP I *n* = 355	*p* value	No ACP II^b^*n* = 85	ACP II *n* = 282	*p* value
**Background characteristics**						
Age at death (years; median, mean)	89, 84	89, 88		89, 88	89, 87	
Gender (n, %):			0.763			0.280
* Female*	7 (58%)	227 (64%)		50 (59%)	184 (65%)	
* Male*	5 (42%)	128 (36%)		35 (41%)	98 (35%)	
Death in NH	9 (75%)	306 (86%)	0.39	68 (80%)	247 (88%)	0.079
Diagnosis (n, %):						
* Cardiovascular disease*	9 (75%)	265 (75%)	1.00	74 (87%)	200 (71%)	*0.003*
* Diabetes*	0 (0%)	72 (20%)	0.13	20 (24%)	52 (18%)	0.300
* Dementia*	7 (58%)	218 (61%)	1.00	34 (40%)	191 (68%)	*<0.001*
* Pulmonary disease*	0 (0%)	51 (14%)	0.39	11 (13%)	40 (14%)	0.771
* Cancer disease*	3 (25%)	44 (12%)	0.19	11 (13%)	36 (13%)	0.966
* Stroke*	1 (8%)	81 (23%)	0.31	22 (26%)	60 (21%)	0.372
* Psychiatric disease*	5 (42%)	127 (36%)	0.76	24 (28%)	108 (38%)	0.090
* Kidney failure*	2 (17%)	61 (17%)	1.00	23 (27%)	40 (14%)	*0.006*
**Physician attendance**						
Number of physician consultations at NH during the patient’s final six months of life (median (range), mean (SD))	1 (0–3),	2 (0–10),	*0.003*	1 (0–10),	2 (0–10),	0.708
	1 (1.0)	2.2 (1.9)		2 (1.9)	2.2 (1.9)	
**EOL care**						
Family members informed of the patient’s deterioration	0 (0%)	296 (83%)	*<0.001*	41 (48%)	252 (89%)	*<0.001*
Family members informed of the patient’s impending death	0 (0%)	282 (79%)	*<0.001*	40 (47%)	241 (85%)	*<0.001*
ED visits during the patient’s final six months of life (median (range), mean (SD))	0 (0–1),	0 (0–4),	0.675	0 (0–2),	0 (0–4),	0.452
	0.3 (0.5)	0.4 (0.7)		0.5 (0.6)	0.4 (0.7)	
Inpatient care occasions during the patient’s final six months of life (median (range), mean (SD))	0 (0–1),	0 (0–3),	0.606	0 (0–2),	0 (0–3),	0.317
	0.3 (0.5)	0.4 (0.6)		0.4 (0.6)	0.3 (0.6)	
Prescription of palliative drugs for symptom relief	5 (42%)	331 (93%)	*<0.001*	66 (78%)	270 (96%)	*<0.001*
‘Benefit for care of closely related person’ certificate^c^	0 (0%)	64 (18%)	0.137	10 (12%)	54 (19%)	0.116

Pearson’s chi-square test was used for group comparisons for categorical data. Fischer’s exact test was used when the expected number was less than five in any cell. *p* values <0.05 were considered significant. Mann Whitney U-test was used for group comparisons of continuous variables because the data was not considered to be normally distributed. Two different definitions of ACP were used: ^a^*ACP I* was defined as ‘a proactive plan to handle a future deterioration of the patient, not only to handle a current, specific problem or situation’. ^b^*ACP II* included the definition of ACP I, the patient’s preferences concerning medical treatment and care (when cognitive impairment was not a limitation), and the involvement of family members. ^c^An employee’s right to be off work in order to attend a closely related person who is seriously ill and receive financial compensation through Swedish social insurance (In Swedish: *Närståendepenning*).

## Discussion

### Statement of principle findings

This study revealed a high prevalence of advance care plans (ACP I: 97% and ACP II: 77%) in nursing home patients. Care plans were more often present in patients with dementia, and less often present in patients with cardiovascular disease and kidney failure. Patients having higher frequency of physician consultations more often had a care plan. Positive associations were seen between the presence of advance care planning and prescription of palliative drugs for symptom relief and family members being given information of the patient’s deterioration and impending death. When hospital care limitations were present in the care plan, both ED visits and periods of inpatient care were seen less frequently during the patient’s final six months of life.

### Strengths and weaknesses

To our knowledge, this is the first retrospective chart review in a nursing home context, using two different advance care plan definitions, of which one is consensus-based [19], strengthening the content validity. The fairly large study population, including individuals from 22 different nursing homes (ordinary long-term homes as well as special units for patients with dementia), supports the generalisability of the findings. This study presents the situation in two counties in Sweden. Although there are national similarities in health care, for example, through national guidelines, regional routines and work documents may contribute to disparities, and internationally, there are also organisational differences in the palliative care and nursing home care. Therefore, the generalisability of the results outside the Swedish counties being studied is partly limited.

There are some additional limitations to this study. Firstly, the retrospective chart review was performed by one researcher, a methodological circumstance that may raise questions about reliability and subjectivity. However, as described, a limited proportion of the health records was also independently reviewed by one of the co-authors, and the results were compared to make sure there was consensus in the interpretation and understanding of the findings. Secondly, we did not collect data regarding when the hospital care limitations in advance care plans were documented in relation to ED visits or periods of inpatient care. Therefore, the actual numbers of ED visits and periods of inpatient care that occurred in spite of hospital care limitations present in the plan might possibly be even lower than the numbers we present. We did not collect data regarding social situation of the patient, i.e. whether the patient had family members or not. This means that advance care plans that fulfilled ACP I criteria also presenting patient preferences concerning medical treatment and care (one of the ACP II criteria), but not the involvement of family members in the care planning (the other ACP II criterion), remained classified as ACP I. This could be seen as a limitation and the prevalence of ACP II may have been higher than presented in our results. Another limitation is that we did not register physician and care characteristics, e.g. whether the patient had a personal physician, the level of continuity of care, the length of the patient-physician relation, age and gender of the physician, etc. Finally, we do not know how many deceased nursing home patients that were available in total in the two counties of the study.

### Findings in relation to other studies

In contrast to the findings of previous studies, which found advance care plans to be rare [1,8–10,13–17], the present study revealed a high prevalence of care plans being in the vast majority (97%) of the study population. When using the comprehensive definition (ACP II), there was still a high prevalence (77%). As concluded by Flo et al. [18], studies on advance care planning in nursing homes are few, and the usage of different definitions of care planning makes the comparison of different study results complex. Adapting these different usages to well-established definitions, such as the EAPC-supported, consensus-based definition of advance care planning [19], would facilitate such comparisons.

The present study showed a great difference in prevalence of care plans restricted to serious respiratory-tract infections in the two counties. In one of the counties (County A), physicians attending nursing homes were encouraged to establish advance care plans with a focus on such serious infections (Covid-19), aiming to prevent this group of patients from being admitted to hospital if unnecessary and/or against the patient’s wishes, and instead to provide these patients with symptom management and good palliative care in the nursing homes. In County B, no encouragement to advance care plans concerning serious respiratory-tract infections was seen, and the prevalence of such plans were therefore particularly fewer compared to those in County A. However, as care plans limited to specific problems or situations were not considered to be according to the definitions of this study, these restricted care plans are not discussed further.

One important reason for performing advance care planning is to proactively establish the principles of autonomy, beneficence and non-maleficence, i.e. health care should be in accordance to patient consent and values, and provide relief from suffering and not cause the patient harm through investigations or treatments that are not considered to benefit the patient. According to Swedish law, the physician is obliged to make decisions on medical care and treatment limitations when appropriate [25], and proactive plans should preferably be based on the patient’s preferences, and/or on the ambition to do good rather than harming the patient. Therefore, advance care planning should be offered when the patient is still capable of participating [26], in order to respect the patient’s autonomy through informed consent and shared decision making. In this study, dementia patients more often had a care plan, and patients with cardiovascular disease and kidney failure more seldom. Reasons for these differences might be physician perceptions that patients with dementia seldom benefit from hospital care and more often are identified as being near EOL than patients with cardiovascular or kidney failure, or it being easier to plan for patients without having to discuss sensitive topics as EOL care. The prevalence of dementia diagnosis was high (*n* = 225; 61%). Similar proportions have been found previously [27]. Individuals with dementia have often lost their ability to understand and make statements concerning treatment decisions. Therefore, initiating advance care planning discussions at an early phase seems important [18,27], in particular concerning patients with dementia moving into a nursing home. In the present study, median time from move into a nursing home to first advance care planning was eight months for the total study population. These months may for some patients with dementia be the difference between being able to participate in such important discussions, for example, regarding preferences concerning treatment, and being too cognitively impaired to participate in discussions and own decision making. If initiating advance care planning discussions when the patient is too cognitively impaired, it is essential to explore what family members know about the patient’s preferences, to ensure that decisions are consistent with what the patient is most likely to have preferred [10].

Reasons for performing an advance care plan/the first care plan in median eight months after admittance to the nursing home, which seems to be a long time, could be that the physician is waiting for the patient to deteriorate, be closer to death, or needs of palliative care. Other reasons could be time aspects as performance of advance care plans requires time (preparation, appointment, documentation, follow-up etc.). Physicians may also find EOL discussions difficult and are reluctant in performing care plans. Most of the nursing home patients in the present study had their last care plan documented during their last month of life (median time 0 months). However, the range was 0–37 months, which means that in extreme cases, the last care plan was documented three years before the patient’s death. Since most deaths in nursing homes are expected deaths, a deterioration of the patient’s condition is probable and therefore a recurrent revision of the care plan would be appropriate to be able to meet the patient’s care needs. Why revision was not done is unknown, but perhaps periodic reminders or best before dates would facilitate or encourage care plan revision.

According to Fosse et al., both nursing home patients and family members underline the importance of physicians being more involved in EOL care [28]. Physicians at nursing homes have been described as absent [28–30] and according to family members there is lack of communication between nursing home staff and the physician [28,29]. The present study showed that advance care plans were positively associated with physician consultations at nursing homes during the patient’s final six months of life. This study did not collect longitudinal data. Therefore, we do not know if a care plan increases the number of physician consultations or vice versa. Since physicians are responsible for performance of the advance care planning, and only physicians can make decisions concerning limitations to life-sustaining care, physician competence and attendance at nursing homes should be seen as central in planning care for nursing home patients.

In this study, patients with advance care plans more frequently received prescriptions of palliative drugs for symptom relief prior to death, and the family members were informed to a greater extent about the patient’s deterioration and impending death compared to those where advance care plans were missing. When creating a care plan, the patient’s impending death is brought into focus, and therefore more extensive preparations for EOL care are made. Prescription of palliative drugs for symptom relief enhances the possibility of good symptom control at EOL, and is a vital component of good palliative care, as well as information being communicated to family members. Indicators of the quality of EOL care are often presented in terms of adequate symptom management, involvement of family members and prevalence of ‘benefit for care of closely related person’ certificate. Encouraging advance care planning in nursing homes could be one important approach to support such good palliative care.

The finding that ED visits and periods of inpatient care were significantly less frequent among individuals with plans to receive all or most care at the nursing home, also known as hospital care limitations, compared to those for whom such limitations were missing, supports the statement that there is adherence to the content of the care plan. However, considering the long residencies with a range of 0–140 months in the study population, it might be probable that performance of an advance care plan with hospital care limitations should have a higher priority in dying patients, than in more stable nursing home patients, who may benefit from such care options outside the nursing home.

Even among individuals with hospital care limitations, the frequency of ED visits and inpatient care during the patient’s final six months of life was surprisingly high, approximately 25% and 20% respectively (Table 3). Uncertainty about prognosis, or how to handle situations and fear of being accused of maleficence could be important reasons for non-adherence to advance care plans when acute situations occur, and the physician needs to make quick decisions regarding the direction of care [31]. Other reasons could be a lack of equipment or staff competence to offer the patient symptom relief. The usage of different health record systems for physicians and nurses working in nursing homes in Sweden could complicate the situation. Documenting in linear form rather than using templates for advance care plans in health records, could hinder finding the care plans in acute situations when quick decisions need to be made regarding content and direction of care.

### Meaning of the study

The results of this study have implications for staff caring for elderly and frail patients, especially nursing home patients. A care plan in the patient’s health record facilitates the physician in making decisions concerning the direction of care when there is a deterioration in the patient’s condition, e.g. to shift focus, when appropriate, from saving life to palliative care. However, the usage of different health record systems and writing in linear form rather than using templates could hinder adherence to the care plan because of difficulties in finding the plans in acute situations when quick decisions regarding direction of the care are needed to be made. In this study, prescriptions of palliative drugs for symptom relief prior to death, were twice as common among patients with advance care planning, compared to those without. Besides the principles of beneficence and non-maleficence, as well as respecting the patient’s autonomy, advance care planning is central to enabling family members to prepare themselves for the impending deterioration and death of the patient [30]. It seems important to support healthcare staff to both initiate advance care planning in nursing home patients, and involve the patient and family members in the care planning and preparation of EOL care.

## Appendix

### Data collection protocol

#### Background characteristics

Participant number/code

Sex (0 = male. 1 = female)

Age at death (years)

Place of death (0 = nursing home. 1 = hospital. 2 = other place.)

Nursing home (name of nursing home)

Length of stay at nursing home (months)

Diagnoses (Cardiovascular disease: y/n, Diabetes: y/n, Dementia: y/n, Pulmonary disease: y/n, Cancer disease: y/n, Stroke: y/n, Psychiatric disease: y/n, Kidney failure: y/n)

#### Acp

ACP documented in medical record (‘breakpoint decision’ included) (0 = no. 1 = ACP restricted to serious respiratory-tract infections (Covid-19) ONLY. 2 = ACP according to ACP I or ACP II. 3 = ACP according to ACP I or ACP II AND ACP restricted to serious respiratory-tract infections (Covid-19).

‘Breakpoint decision’ since moving into NH (0 = no. 1 = yes, templates being used. 2 = yes, written in linear form.)

Time for documented ‘breakpoint decision’? (days before death)

Time from move into NH to first ACP (months)

Number of ACPs, ‘breakpoint decision’ included (n)

Time from last ACP to death (months)

Time from moving into NH to first ACP (months)

Patient’s preferences (0 = preferences missing, no cognitive impairment. 1= preferences present. 2= preferences missing, cognitive impairment.)

Family members participating in ACP, physically or by phone (y/n)

Family members informed of ACP content (y/n)

#### Care limitations in ACP

Do-not resuscitate (DNR) order (0 = not present. 1= present)

Hospital care limitation (0 = not present. 1= patient should never receive hospital care. 2 = hospital care only in case of fracture, acute chest pain etc.)

#### EOL care

Number of physician consultations at NH during the patient’s final six months of life (n)

Numbers of ED visits during the patient’s final six months of life (n)

- Time for last ED visit (months before death)
- Reason for last ED visit (documented reason according to physician at ED)

Inpatient care occasions during the patient’s final six months of life (n)

- Time for last inpatient care occasion (months before death)
- Reason for last inpatient care occasion (documented reason according to physician at hospital)

Prescription of palliative drugs for symptom relief (y/n)

Family members informed of the patient’s deterioration (y/n)

Family members informed of the patient’s impending death (y/n)

“Benefit for care of closely related person” certificate (y/n)
